# Supplementary material for: Typical 2-Cys Peroxiredoxins as a Defense Mechanism against Metal-Induced Oxidative Stress in the Solitary Ascidian Ciona robusta
Source: Antioxidants (Basel). 2021 Dec 30;11(1):93. doi: 10.3390/antiox11010093 (PMC8772837; doi:10.3390/antiox11010093)
Supplement: Supplementary file 1 [file antioxidants-11-00093-s001.zip › antioxidants-1509845-supplementary.pdf]

SUPPLEMENTARY MATERIAL

Tables

Table S1. Primer sequences, and relative melting temperatures (T_m), used for reverse transcription (Oligo-dT Anchor primer) and for PCR amplification.

Primers	Sequences 5'-3'	Tm (°C)
Oligo-dT Anchor	ACCACGCGTATCGATGTCG(T)16	75.2
CrPrdx2fw	AGTCCGCCCCAGATTTTA	56.8
CrPrdx2rv	CCGTGCTGGTCTGTGAAC	57.6
CrPrdx3fw	TACACGGGTTTCGACGGA	57.0
CrPrdx3rv	GATCCTGGTGTCCAACTAG	56.4
CrPrdx4fw	GCTTGTTCCTTGCTCACA	55.0
CrPrdx4rv	AGACCTCACCATGCTCAT	54.4
Crβactfw	TGATGAAGATCTTGACCGAG	54.6
Crβactrv	CTTGTTTGCTGATCCACATC	55.2

Table S2. Primer sequences, and relative melting temperatures (T_m), used for qRT-PCR amplification.

Primers	Sequences 5'-3'	Tm (°C)
CrPrdx2fw_RT	CCGCCCCAGATTTTACGGCAA	63.3
CrPrdx2rv_RT	GCACCTCACACCCAATATCAC	62.5
CrPrdx3fw_RT	CGGGTTTCGACGGACACATAC	61.5
CrPrdx3rv_RT	CTTTGAATGGTGGAGCAGGC	60.6
CrPrdx4fw_RT	TCTGAGGGAGGTCTTGGAAA	60.1
CrPrdx4rv_RT	AGAGCCCACGAAGAGTGTGT	60.0
Crβactfw_RT	CCTTGGTATGGAATCTGCTG	62.1
Crβactrv_RT	GAGAGAACGGTGTTGGCGTA	65.2

Table S3. GenBank accession numbers and ANISEED transcript IDs (*Ciona robusta, Ciona intestinalis* and *Botrylloides leachii*) referred to amino acid sequences of Prdx2, Prdx3 and Prdx4 used for multi-alignment and phylogenetic analyses.

Species	Prdx2	Prdx3	Prdx4
Ciona robusta	KY.Chr8.1384.v1.nonSL16	KY.Chr10.1364.v1.ND1	KY.Chr10.1036.v1.SL1
Ciona intestinalis	KH.L17.4.v1.A.nonSL9	KH.S396.2.v1.A.nonSL5	KH.C10.328.v1.A.SL1
Botrylloides leachii	Boleac.CG.SB_v3.S416	Boleac.CG.SB_v3.S163	Boleac.CG.SB_v3.S416
Danio rerio	NP_001002468.1	NP_001013478.3	NP_001082894.1
Oryzias latipes	XP_011478972.1	XP_023819390.1	XP_004076406.1
Mylopharyngodon piceus	ALD62538.1	ALD62539.1	ALD62540.1
Callorhinchus milii	XP_007905343.1	XP_007903837.1	NP_001279887.1
Xenopus laevis	AEM44539.1	AEM44540.1	AEM44541.1
Rhinatrema bivittatum	XP_029440999.1	XP_029466379.1	XP_029459262.1
Chelonia mydas	XP_037743763.1	XP_037761793.1	XP_037757601.1
Python bivittatus	XP_007421035.1	XP_007429204.1	XP_007436716.1
Podarcis muralis	XP_028573219.1	XP_028585929.1	XP_028583327.1
Anas platyrhynchos		XP_027316512.2	XP_027304862.1
Lepidothrix coronate		XP_017664753.1	XP_017682647.1
Tupaia chinensis	XP_006161057.1	XP_006160463.1	XP_027626915.1
Myotis myotis	XP_036158570.1	XP_036191702.1	XP_036161216.1
Mus musculus	NP_035693.3	NP_031478.1	NP_001300640.1
Vicugna pacos	XP_006206304.1	XP_006200224.1	XP_006213064.1
Bos indicus	XP_019819615.1	XP_019808318.1	XP_019811886.1
Equus caballus	XP_023501083.1	XP_001493666.1	XP_023489068.1
Canis lupus	XP_038284392.1	NP_001243414.1	XP_038305572.1
Homo sapiens	NP_005800.3	ABB84468.1	NP_006397.1
Macaca mulatta	NP_001248186.1	NP_001252755.1	NP_001180740.1
Clonorchis sinensis	AEK86199.1		
Schistosoma haematobium		XP_012798879.1	
Drosophila melanogaster		NP_524387.1	
Amphioctopus fangsiao			AGF80254.1
Sepiella maindroni			AKN23454.1
Crassostrea gigas	XP_011441532.1		
Aplysia californica	XP_005089483.1		
Scylla paramamosain		ASS34530.1	ASS34531.1
Culex pipiens	XP_001852614.1		

Table S4. Percentages of identity, similarity and *E*-values obtained by comparing Cr-Prdx2 amino acid sequence with orthologous sequences of metazoans.

Species	% identity	% similarity	*E*-values
Danio rerio	74.6	90.4	5.8e-84
Python bivittatus	74.6	88.8	4.7e-81
Mus musculus	71.1	92.4	5.7e-87
Homo sapiens	70.6	89.8	1.0e-87
Crassostrea gigas	70.6	88.7	3.3e-81
Xenopus laevis	67.5	90.4	9.1e-80
Botrylloides leachii	66.3	87.8	1.3e-72
Culex pipiens	65.5	88.7	7.6e-76
Clonorchis sinensis	59.9	83.4	2.4e-67

Table S5. Percentages of identity, similarity and *E*-values obtained by comparing Cr-Prdx3 amino acid sequence with orthologous sequences of metazoans.

Species	% identity	% similarity	*E*-values
Xenopus laevis	72.4	89.2	8.1e-82
Danio rerio	71.8	88.3	3.8e-85
Python bivittatus	71.3	88.6	5.6e-82
Anas platyrhynchos	70.4	89.7	1.5e-84
Mus musculus	70.0	88.3	2.0e-75
Scylla paramamosain	67.0	86.7	5.2e-79
Drosophila melanogaster	66.7	90.1	4.3e-78
Homo sapiens	66.7	87.2	2.0e-74
Botrylloides leachii	64.7	88.9	9.1e-51
Schistosoma haematobium	64.6	85.4	3.3e-79

Table S6. Percentages of identity, similarity and *E*-values obtained by comparing Cr-Prdx4 amino acid sequence with orthologous sequences of metazoans.

Species	% identity	% similarity	*E*-values
Botrylloides leachii	88.9	96.6	8.6e-100
Danio rerio	78.2	91.3	4.1e-102
Homo sapiens	77.9	91.5	3.3e-88
Xenopus laevis	77.2	91.6	2.3e-94
Anas platyrhynchos	76.7	90.6	6.4e-90
Sepiella maindroni	76.6	92.5	9.0e-98
Python bivittatus	75.5	91.2	4.1e-85
Mus musculus	74.6	90.6	6.6e-79
Scylla paramamosain	67.8	85.5	2.0e-90

Figures

```
1     gtt ata atc atg tcc gcc ggt aaa gct tgc att caa aag tcc gcc cca gat ttt acg gca
                   M   S   A   G   K   A   C   I   Q   K   S   A   P   D   F   T   A
61    aca gca gtt gtg aac gga gat ttt cgt gat att tca cta agc gaa tat aaa gga aaa tat
       T   A   V   V   N   G   D   F   R   D   I   S   L   S   E   Y   K   G   K   Y
121   gtt gtt ttg ttt ttt tac ccg ctc gac ttc acg ttc gtt tgc ccg acg gag atc att gcg
       V   V   L   F   F   Y   P   L   D   F   T   F   V   C   P   T   E   I   I   A
181   ttt agt gat cgt gtg tca gag ttc cgt gat att ggg tgt gag gtg ctc gcg tgt tcc aca
       F   S   D   R   V   S   E   F   R   D   I   G   C   E   V   L   A   C   S   T
241   gac tca cac ttc agt cat ctc gca tgg aca aac atc cca agg aag aag ggg gga atc gga
       D   S   H   F   S   H   L   A   W   T   N   I   P   R   K   K   G   G   I   G
301   aac atg aaa atc ccg ttg att gcc gac aaa aac tgt gca ata tct aaa gac tac ggg gtt
       N   M   K   I   P   L   I   A   D   K   N   C   A   I   S   K   D   Y   G   V
361   ttg atg gaa ggc agt gga atc gcg ttc agg gga ttg ttc atc atc gat acc atg gga att
       L   M   E   G   S   G   I   A   F   R   G   L   F   I   I   D   T   M   G   I
421   ctt cgg caa ata acg att aat gat ttg cct gtc ggt cgc tca gtg gac gag acg cta cga
       L   R   Q   I   T   I   N   D   L   P   V   G   R   S   V   D   E   T   L   R
481   ttg gtc aaa gcg ttt cag ttc aca gac cag cac ggc gag gtt tgc cct gct ggg tgg aaa
       L   V   K   A   F   Q   F   T   D   Q   H   G   E   V   C   P   A   G   W   K
541   ccc ggc gat gat acg atc aaa cca gat gtt caa gat agc cag aaa tat ttc agt aaa caa
       P   G   D   D   T   I   K   P   D   V   Q   D   S   Q   K   Y   F   S   K   Q
601   taa aca ccc ctg tga ata ttt cag cat cgt tta ttt aca ttt att aca aag gca gta gtg
       -
661   ttt aat
```

Figure S1. Cr-Prdx2 cDNA sequence and relative amino acid sequence; 5'- and 3'-UTR regions are underlined; in grey sequence obtained with the amplicon sequencing; degradation signals are boxed in bold.

```
1     gtc ata cat gcg cag taa caa cac gaa acg aag aaa att cac ttt tct ata ttc taa cag
61    aat ttc ttt ctg taa tgt ttt gac ttc tgt taa tta gaa tat aca aat agt tat tac atc
121   atg ttt att aat aga ttg gta cac ggg ttt cga cgg aca cat aca ttg cag tcc agt ctt
       M   F   I   N   R   L   V   H   G   F   R   R   T   H   T   L   Q   S   S   L
181   aac cca tgg atc tca aaa tgg aat cca aac tca gcc agt att tca aca tca tgc aga tcc
       N   P   W   I   S   K   W   N   P   N   S   A   S   I   S   T   S   C   R   S
241   atg gtt gcc cag gta aca cag cct gct cca cca ttc aaa ggt atg tcc gtg gta gaa gga
       M   V   A   Q   V   T   Q   P   A   P   P   F   K   G   M   S   V   V   E   G
301   aaa ttc aag gaa ata tca ctt gaa gat tac aag gga aaa tat ttg gtt ctc ttc ttt tac
       K   F   K   E   I   S   L   E   D   Y   K   G   K   Y   L   V   L   F   F   Y
361   cct ctt gat ttc act ttt gtt tgc ccc act gaa atc atc tca ttc agc gac aaa agt cca
       P   L   D   F   T   F   V   C   P   T   E   I   I   S   F   S   D   K   S   P
421   gag ttt gaa aaa ctt gac aca gcg gtt gta gga gcc tca gtt gac tct cac ttc agc cat
       E   F   E   K   L   D   T   A   V   V   G   A   S   V   D   S   H   F   S   H
481   ctt gcg tgg ata aat aca cct agg aaa caa ggg gga ctt gga gaa atg aaa ata ccc ctg
       L   A   W   I   N   T   P   R   K   Q   G   G   L   G   E   M   K   I   P   L
541   ctt tcc gat ctt act aaa aat ata tca aga gat tat ggg gtg ttg ctg gag aat gct ggg
       L   S   D   L   T   K   N   I   S   R   D   Y   G   V   L   L   E   N   A   G
601   att gca ttg aga ggg ttg ttc ata att gat cca tcc ggc acc ata agg cac gcg agc gtt
       I   A   L   R   G   L   F   I   I   D   P   S   G   T   I   R   H   A   S   V
661   aat gat ttg cct gtt gga agg tct gtt gat gaa gtt ttg agg ttg gtt caa gct ttc cag
       N   D   L   P   V   G   R   S   V   D   E   V   L   R   L   V   Q   A   F   Q
721   ttt gtt gac aaa cat ggg gag gtg tgc cct gct agt tgg aca cca gga tca aaa act atc
       F   V   D   K   H   G   E   V   C   P   A   S   W   T   P   G   S   K   T   I
781   aaa cca agt gtg gat ggt tca aag aca tat ttt gaa gag gca aat taa aac aca cat act
       K   P   S   V   D   G   S   K   T   Y   F   E   E   A   N   -
841   gtc aac tat cat gta caa att gta ttt att ttt tgg gta caa atc gca cgc att taa tct
901   ttt ggt tgt att caa taa ata tac tgt gta ctc taa tgt aca caa ttt att tat ttg gtt
961   gta tct aat aaa tca att taa tac aac ata act att tag tat tta tta ttc ttt tta atc
1021  aca gca gcg aag acc aaa ttc att aaa aca atg aag aga agg aaa tta gca gca aaa taa
1081  cag tta tta aaa cct agt tta att gca aaa ttt tgg cga ccc aat ttc cga aac tac att
1141  tca cac aac ttt tgt aag cat gaa ata aaa tga ttt tac ttt tac aat
```

Figure S2. Cr-Prdx3 cDNA sequence and relative amino acid sequence; 5'- and 3'-UTR regions are underlined; in grey sequence obtained with the amplicon sequencing; polyadenylation signals are boxed in double line; degradation signals are boxed in bold.

```
1     gct tct tag ttt ttg tgt ata att agg ctt ttc gtt tga tct gca ttg att tgc gca aac
61    atg gga gtc tca ttt caa ctt ttc act ctt gca tgc ttg ttc ctt gct cac agt gga ata
       M   G   V   S   F   Q   L   F   T   L   A   C   L   F   L   A   H   S   G   I
121   tgc caa gaa cgt gct gtt ttc tgg cca cca tat gaa agt cac ttg ctg caa tct acc aaa
       C   Q   E   R   A   V   F   W   P   P   Y   E   S   H   L   L   Q   S   T   K
181   gct caa atc tcg aaa cca gcg cca gac tgg gag ggt aca gcc att gtc gat ggt gaa att
       A   Q   I   S   K   P   A   P   D   W   E   G   T   A   I   V   D   G   E   I
241   aaa aca ata aag ctg gga gat tac aaa gga aaa tac ctt att ttc ttt ttt tac cct ctt
       K   T   I   K   L   G   D   Y   K   G   K   Y   L   I   F   F   F   Y   P   L
301   gat ttc act ttt gta tgt cca act gag atc att gcc ttc tca gac agg gtt gct gag ttt
       D   F   T   F   V   C   P   T   E   I   I   A   F   S   D   R   V   A   E   F
361   aag aaa att aat gct gaa gtt gtg gca gct tct gtt gat tct cac ttc acc cat ctc gca
       K   K   I   N   A   E   V   V   A   A   S   V   D   S   H   F   T   H   L   A
421   tgg tta aac acc cat cgc tct gag gga ggt ctt gga aaa ctc aac att cct ctt ctg tca
       W   L   N   T   H   R   S   E   G   G   L   G   K   L   N   I   P   L   L   S
481   gat ctc act cat aag ata tca agg gat tat gga gtc ctt cta gag gac ctc gga cac act
       D   L   T   H   K   I   S   R   D   Y   G   V   L   L   E   D   L   G   H   T
541   ctt cgt ggg ctc ttc atc att gat ccc aaa ggt atc ctg aga cag atc act atg aat gat
       L   R   G   L   F   I   I   D   P   K   G   I   L   R   Q   I   T   M   N   D
601   ctt cca gtt gga aga tct gta gac gag act ttg aga ttg gtc caa gca ttc caa cat act
       L   P   V   G   R   S   V   D   E   T   L   R   L   V   Q   A   F   Q   H   T
661   gat gag cat ggt gag gtc tgt cct gct ggt tgg gag cca ggt aaa gac acc att att cca
       D   E   H   G   E   V   C   P   A   G   W   E   P   G   K   D   T   I   I   P
721   gac ccc aag gat aaa ctg aag tac ttc cac aaa aca tca aaa gcc aag ggt gaa ttg taa
       D   P   K   D   K   L   K   Y   F   H   K   T   S   K   A   K   G   E   L   -
781   act gtt gaa atg tca tct gtg tct tgt ttt att ggt tgt tgt tgt ttg tag ggt gtg tgt
841   ttg ttt aag atg aag cag ctg tag ggt tat cag tgc aat ttc aag aaa gaa ata gtt taa
901   tca ctt taa tgt gtc tgt ttt caa gaa agc aat agt tta atc cct tta agt gtc tgt taa
961   aag ctg tgc gta ttt atc tta aac ttt tgt aaa att tag cag cct tta aat tga aaa aaa
1021  aaa ctg ttg tgt ttg ttg cta tta tct aat tgt gcc aaa gtt aag ttt ctg tac caa aat
1081  tat att gta att cat ttt cta aaa caa ttg tgt cat gtt ctg caa ttt aat tgt gtc gga
1141  ctg tcg gtt acc tgt atg tgt aat aaa gca atg ttt ttg cga ttt tgt tgg tca tgt atc
1201  gta tgt gtg gac taa aat gtg ttt atg ttt cgt ttc aac
```

Figure S3. Cr-Prdx4 cDNA sequence and relative amino acid sequence; 5'- and 3'-UTR regions are underlined; in grey sequence obtained with the amplicon sequencing; polyadenylation signals are boxed in double line; degradation signals are boxed in bold.

```
Clonorchis sinensis              ----------------------------------------------------MALLPNQP      8
Culex pipiens quinquefasciatus   ------------MSFATKALLRNIPQLS-KLASSGTV----QKNLFHTARALSVAQVQKP      43
Botrylloides leachii             -------MKLFIICFIIVTVLANDEEQKYGGMP-GMFWPP----YQSHLLQSTKAQISKP      48
Crassostrea gigas                MASSVNMLFFGIVCLIACTFCRAADDDKCQSFAGGSVFPQETRRTSGHAIQWSQAVISKP      60
Ciona robusta                    -------------------------------------------------MSAGKACIQKS      11
Xenopus laevis                   ---------------------------------------------MACPVRAVKTHIGKP      15
Homo sapiens                     -------------------------------------------------MASGNARIGKP      11
Mus musculus                     -------------------------------------------------MASGNAQIGKS      11
Danio rerio                      -------------------------------------------------MSAGNAKIGQP      11
Python bivittatus                -------------------------------------------------MASGKAHIGKP      11
                                                                                           :

Clonorchis sinensis              APEFSGMAVVNGEFKNISLKDYRGKYVILLFYPLDFTFVCPTELIAFSDAAEEFKSKNCV      68
Culex pipiens quinquefasciatus   APPFSGTAVVNNDFKEIKLADFKGKYLVLFFYPLDFTFVCPTEIIAFSDRIKDFHALNAE      103
Botrylloides leachii             APSWEGTAIVDGEIKTIKSDDYLGKYLIFFFYPLDFTFVCPTEIIAFSDRAEEFRKIGAE      108
Crassostrea gigas                APDFNGTAVIKGEFKDIKLSDYKGKYLVFFFYPLDFTFVCPTEIIAFSDRVGEFKKINTE      120
Ciona robusta                    APDFTATAVVNGDFRDISLSEYKGKYVVLFFYPLDFTFVCPTEIIAFSDRVSEFRDIGCE      71
Xenopus laevis                   SPAFQATALVNGEFKEIQLSDYLGKYVVLFFYPLDFTFVCPTEIIAFSNHAEDFKKINCQ      75
Homo sapiens                     APDFKATAVVDGAFKEVKLSDYKGKYVVLFFYPLDFTFVCPTEIIAFSNRAEDFRKLGCE      71
Mus musculus                     APDFTATAVVDGAFKEIKLSDYRGKYVVLFFYPLDFTFVCPTEIIAFSDHAEDFRKLGCE      71
Danio rerio                      APQFKATAVVDGQFKDIQLSDYRGKYVVLFFYPLDFTFVCPTEIIAFSERAAEFRKIGVE      71
Python bivittatus                VPDFHATAVVDGTIKDIKLSDYRGKYVVLFFYPLDFTFVCPTEIIAFNDRVEEFRKINCE      71
                                  * : . *::.. :: :.  :: ***::::*************:***.:   :*:  .

Clonorchis sinensis              IIGCSTDSVYAHLQWTKMDRKVGGLGKMNIPLLSDKNMKISRAYHVLDEEEGHAFRGQFL      128
Culex pipiens quinquefasciatus   VVGVSVDSHFSHLAWCNTPRKSGGLGKLEYPLLADLTKKISADYGVLLEEAGISLRGLFI      163
Botrylloides leachii             VVACSVDSHFTHLAWTNTERKEGGLGKINIPLLSDLTHKISRDYGVLLEDLGHTLRGLFI      168
Crassostrea gigas                VIACSVDSQFTHLAWINTPRAKGGLGPMNIPLLSDITHEISKAYGVYLQDLGHSLRGLFI      180
Ciona robusta                    VLACSTDSHFSHLAWTNIPRKKGGIGNMKIPLIADKNQAISKDYGVLMEGSGIAFRGLFI      131
Xenopus laevis                   LIAVSVDSQFTHLAWTKVPRKEGGLGPVNIPLVSDLTHSIAKDYGVLKEEDGVAYRGLFI      135
Homo sapiens                     VLGVSVDSQFTHLAWINTPRKEGGLGPLNIPLLADVTRRLSEDYGVLKTDEGIAYRGLFI      131
Mus musculus                     VLGVSVDSQFTHLAWINTPRKEGGLGPLNIPLLADVTKSLSQNYGVLKNDEGIAYRGLFI      131
Danio rerio                      LIAASTDSHFSHLAWINTPRKQGGLGSMNIPLVADLTQSISRDYGVLKEDEGIAYRGLFV      131
Python bivittatus                VIAASVDSQFTHLAWINTSRKEGGLGSTNIPLVADINHNISKDYGVLKEEDGIAYRGLFI      131
                                 ::. *.** ::** * :  *  **:*  : **::* .  ::  * *     * : ** *:

Clonorchis sinensis              IDPKGVLRQITVNDRPVGRSVEEAIRLLEAFHFHEQHGEVCPAKWKPK--GKTMKADPVA      186
Culex pipiens quinquefasciatus   IDPNGVVRQVTINDLPVGRSVDETLRLIKAFQFVEKHGEVCPANWDPKANADTIKPDPKG      223
Botrylloides leachii             IDPKGILRQITMNDLPVGRSVDETLRLVQAFQHTDQHGEVCPAGWQPG--SDTIIPDPEG      226
Crassostrea gigas                IDPKGTLRQITMNDLPVGRSVDETLRLVQAFQYTDTHGEVCPAGWKPG--SDTIIPDPKE      238
Ciona robusta                    IDTMGILRQITINDLPVGRSVDETLRLVKAFQFTDQHGEVCPAGWKPG--DDTIKPDVQD      189
Xenopus laevis                   IDGKGILRQITINDLPVGRSVDETLRLVQAFQYTDVHGEVCPAGWKPG--SSTIKPNVKD      193
Homo sapiens                     IDGKGVLRQITVNDLPVGRSVDEALRLVQAFQYTDEHGEVCPAGWKPG--SDTIKPNVDD      189
Mus musculus                     IDAKGVLRQITVNDLPVGRSVDEALRLVQAFQYTDEHGEVCPAGWKPG--SDTIKPNVDD      189
Danio rerio                      IDDKGILRQITINDLPVGRSVDETLRLVQAFQHTDKYGEVCPAGWKPG--SDTIVPDVQK      189
Python bivittatus                IDDKGILRQITVNDLPVGRSVDETLRLVQAFQFTDQHGEVCPAGWQPG--GDTIKPTVKD      189
                                 **  * :**:*:** ******:*::**::**:. : :****** *.*    .*:

Clonorchis sinensis              AQEYFSSVN--    195
Culex pipiens quinquefasciatus   SKEYFNKHG--    232
Botrylloides leachii             KMKYFQKMREL    237
Crassostrea gigas                SQKYFSKQNN-    248
Ciona robusta                    SQKYFSKQ---    197
Xenopus laevis                   SKEFFSKEY--    202
Homo sapiens                     SKEYFSKHN--    198
Mus musculus                     SKEYFSKHN--    198
Danio rerio                      SKEFFSKQ---    197
Python bivittatus                SKLFFSKQQ--    198
                                    :*..
```

Figure S4. Schematic Prdx2 domain organization. The AhpC-TSA domain is highlighted in light grey, the 1-cysPrx C domain is highlighted in dark grey. For *C. robusta*, alpha helixes and beta sheets are boxed with dotted and continues lines respectively; amino acids of the catalytic center are indicated in bold and underlined. *= identical amino acids (completely preserved), := very similar amino acids (semi-conservative substitution), .= similar amino acids (conservative substitution); numerals refer to character counts.

```
Botrylloides leachii          -----------------------------------------MATLRSCASLVTKSIAASK  19
Drosophila melanogaster       ----MSF--------V--------AR----------SLIRNVPLMGK---AIL-------  20
Schistosoma haematobium       ---------------------------------------------MLLQVLI-------  7
Ciona robusta                 -----MFINRLVHGFRRTH-----------------------TLQSSLNPWI-------  24
Scylla paramamosain           --------------------MAGL--------------LKNIAV--TATRTVL-------  17
Danio rerio                   MAATIG---RLLGASARGAAVCGLKTLVPRNGASVI----------RAPQPLA-------  40
Python bivittatus             MAALTGVLRRLLKPSSGWT-----GATLWRAGAVAGRCLSGAASGRKCAADA--------  47
Xenopus laevis                MAASCG---RLLASWVGRT-----GRLTGQ-LPRVAG--SSVPRNMTAVTPSI-------  42
Anas platyrhynchos            MAAALG---RLLRSAVPAA-----GRRLQ---------------GR--------------  23
Homo sapiens                  MAAAVG---RLLRASVARH-----VSAIPW-GISATAALRPAACGRTSLTNLL-------  44
Mus musculus                  MAAAAG---RLLWSSVARH-----ASAISR-SISASTVLRPVASRRTCLTDIL-------  44

Botrylloides leachii          QLRLLVANHHLRPLSSSSRNHVAQVTQPAPDF----------------------------  51
Drosophila melanogaster       ---SQQKQIAARLLHQTAPLAAVRVQQPAPDFKGLAVVDNSFQEVKLEDYRGKYLVLFFY  77
Schistosoma haematobium       ---KGALRYNRSPVSNLCRHYAAHVQRPAPDFCGTAVVDGQFKEIKLRDYAGKYLVLFFY  64
Ciona robusta                 ----SKWNPNSASISTSCRSMVAQVTQPAPFFKGMSVVEGKFKEISLEDYKGKYLVLFFY  80
Scylla paramamosain           ---PRITAAAQRGLSTSVRCLAPAVTQPAPSFKAQAVVNGDFKELSLEDFRGKYLVLFFY  74
Danio rerio                   ---C--IAAQKACFSISAARWAPAVTQAAPHFKGTAVINGEFKEISLGDFKGKYLVLFFY  95
Python bivittatus             -----TPLVPRFHFSTSASRFVPAVTQHAPYFKGTAVVDGDFKELSLDDFKGKYLVLFFY  102
Xenopus laevis                ---C---AAQKLQFSTSSGRFLPAVTQHAPQFKGTAVVNGEFKELSLEDFKGKYLVLFFY  96
Anas platyrhynchos            -----PQLLAHRQLSLGASRLAPAVTQHAPFFKGTAVVHGEFKELSLDDFKGKYLVLFFY  78
Homo sapiens                  ---CSGSSQA-KLFSTSSSCHAPAVTQHAPYFKGTAVVNGEFKDLSLDDFKGKYLVLFFY  100
Mus musculus                  ---WSASAQGKSAFSTSSSFHTPAVTQHAPYFKGTAVVNGEFKELSLDDFKGKYLVLFFY  101
                                           .          * : ** *

Botrylloides leachii          ---------------------------------QDSRFSHLASVNTPRSKYGFGDTRIPL  78
Drosophila melanogaster       PLDFTFVCPTEIVAFSERIKEFHDINTEVLGVSVDSHFSHLTWCNVDRKNGGVGQLKYPL  137
Schistosoma haematobium       PLDFTFVCPTELTAFSDRIDEFKNEGVEVVGVSTDSHFSHLAWINTPRKEGGLGCLRYPL  124
Ciona robusta                 PLDFTFVCPTEIISFSDKSPEFEKLDTAVVGASVDSHFSHLAWINTPRKQGGLGEMKIPL  140
Scylla paramamosain           PLDFTFVCPTELIAFSDHMAAFRELNCEVVGVSTDSHFSHLAWNNMPRKQGGLGGLQYTL  134
Danio rerio                   PLDFTFVCPTEIVAFSDKANEFHDVNCAVVGVSVDSHFTHLAWTNTPRKSGGLGKIQIPL  155
Python bivittatus             PLDFTFVCPTEIIAFSDKANEFHDVNCDVLAVSVDSHFCHLAWINTPRKSGGLGHMNIPL  162
Xenopus laevis                PLDFTFVCPTEIVAFSNKANEFHDVNCEVVAVSVDSHFCHLAWTNTPRKSGGLGQMNIPL  156
Anas platyrhynchos            PLDFTFVCPTEIVAFSNKANEFRDVNCEVVAVSVDSHFCHLAWINTPRKSGGLGKMNIPV  138
Homo sapiens                  PLDFTFVCPTEIVAFSDKANEFHDVNCEVVAVSVDSHFSHLAWINTPRKNGGLGHMNIAL  160
Mus musculus                  PLDFTFVCPTEIVAFSDKANEFHDVNCEVVAVSVDSHFSHLAWINTPRKNGGLGHMNITL  161
                                                                **:* **:  *  *.. *.*  .  :

Botrylloides leachii          LSDFNKTISKEYGILVEQAGVALRGTYIIDTNGIVRHLSVNDFSVGRSVDEILRLVQAFQ  138
Drosophila melanogaster       LSDLTKKISADYDVLLDKEGISLRGTFIIDPNGILRQYSINDLPVGRSVDEVLRLIKAFQ  197
Schistosoma haematobium       LADYQKQITRDYGVLQEELGVALRGLFIINPDGIVRQITINDLPVGRSVDEVLRLVRAFQ  184
Ciona robusta                 LSDLTKNISRDYGVLLENAGIALRGLFIIDPSGTIRHASVNDLPVGRSVDEVLRLVQAFQ  200
Scylla paramamosain           LADLNKTISRDYGVLLEDDGVALRGLFLIDPEGVVRHMSINDLPVGRSVEETLRLLKAFQ  194
Danio rerio                   LADLTKQVSRDYGVLLEGPGIALRGLFIIDPNGIVRHMSVNDLPVGRSVEETLRLVKAFQ  215
Python bivittatus             LSDLTKQISRDYGVLIEGSGLALRGLFIIDPNGIIKHLSVNDLPVGRSVEETLRLVKAFQ  222
Xenopus laevis                LSDLNKQISRDYGVLLETAGIALRGLFIIDPNGIIKHMSVNDLPVGRSVEETLRLVKAFQ  216
Anas platyrhynchos            LSDLTKQISRDYGVLLEGPGIALRGLFIIDPNGVIKHLSVNDLPVGRSVEETLRLVKAFQ  198
Homo sapiens                  LSDLTKQISRDYGVLLEGSGLALRGLFIIDPNGVIKHLSVNDLPVGRSVEETLRLVKAFQ  220
Mus musculus                  LSDITKQISRDYGVLLESAGIALRGLFIIDPNGVVKHLSVNDLPVGRSVEETLRLVKAFQ  221
                              *:*  * :: :*.:* :  *::*** ::*: .* ::: ::**: *****:* ***::***

Botrylloides leachii          FXDEHGQLCPSSWKPGS--DSIKPTVEGSKEYFDKIE-     173
Drosophila melanogaster       FVEQHGEVCPANWNPNSNPATIKPDVEESKKYFSKHG-     234
Schistosoma haematobium       FTDKHGEVCPAGWQPKG--PTIKPDLKKYKEYFHKVN-     219
Ciona robusta                 FVDKHGEVCPASWTPGS--KTIKPSVDGSKTYFEEAN-     235
Scylla paramamosain           FVAEHGEVCPANWQPES--PTIKPDPTGSLEYFNKVN-     229
Danio rerio                   FVETHGEVCPASWTPKS--PTIKPTPDGSKEYFEKVN-     250
Python bivittatus             YVETHGEVCPANWIPDS--PTIKPSPEASKEYFAKVNK     258
Xenopus laevis                FVETHGEVCPAXWTPDS--PTIKPSPEGSKDYFEKVH-     251
Anas platyrhynchos            YVETHGEVCPANWTPDS--PTIKPSPEGSKEYFEKVHK     234
Homo sapiens                  YVETHGEVCPANWTPDS--PTIKPSPAASKEYFQKVNQ     256
Mus musculus                  FVETHGEVCPANWTPES--PTIKPSPTASKEYFEKVHQ     257
                              :   **::**: * * .   :***       ** :
```

Figure S5. Schematic Prdx3 domain organization. The AhpC-TSA domain is highlighted in light grey, the 1-cysPrx_C domain is highlighted in dark grey. For *C. robusta*, alpha helixes and beta sheets are boxed with dotted and continues lines respectively; amino acids of the catalytic center are indicated in bold and underlined. *= identical amino acids (completely preserved), := very similar amino acids (semi-conservative substitution), .= similar amino acids (conservative substitution); numerals refer to character counts.

```
Ciona robusta             --------------------------MGVSFQLFTLACLFLA-----------------H      17
Botrylloides leachii      ------------------------------MKLFII-CFIIVTVLAN----------DEE      19
Scylla paramamosain       ---------------------------MSRLNLVLLACLV--------G-------LSVH      18
Sepiella maindroni        ----------------------------MALVGVNILCLLVLI--------------SSA      18
Mus musculus              MEARSK------------LLDGTTASRRWTRKLVLLLPPLLLFLLRTESL--QGLESDER      46
Python bivittatus         MEVGRQARGPPSESQAAARMSGRCGRLKPVSEPLAVYSMVLLMLAATMSEEAGTAEAAQS      60
Danio rerio               MD--VSRC-----------V------KTPREWLGVLWA-LLLL-SESVVC-------DGA      32
Homo sapiens              --MEALPL-----------LAAT-TPDHGRHRRLLLLP-LLLFLLPAGAVQG--WETEER      43
Xenopus laevis            MALQLRRY-----------L---------RGSPAVTLC-LLLLSAAAVTCEEEQSQARPG      39
Anas platyrhynchos        MEAGRRGL-----------RAGT-RTGTGTGGSALLVA-V-LAVLAVCGAAAEAEDPRAA      46
                                                                 .

Ciona robusta             SGICQ------ERAV------FWPPYESHLLQSTKAQISKPAPDWEGTAIVDGEIKTIKL      65
Botrylloides leachii      QKYGG------MPGM------FWPPYQSHLLQSTKAQISKPAPSWEGTAIVDGEIKTIKS      67
Scylla paramamosain       GSLAEEQCHTFAGGAVYPNTEGR--ASGHTLQWTSAMISKPAPTWEGTAVVDGQLQELKL      76
Sepiella maindroni        VCIADEKCYSFAGGQVYPQEDRK--MSGHTIQWSKAIISKPAPDWNGTAVINGKFEEIGL      76
Mus musculus              FRTRENECHFYAGGQVYPGEASRVSVADHSLHLSKAKISKPAPYWEGTAVINGEFKELKL      106
Python bivittatus         YGYGDEKCHYYAGGEVYPGEASRVPVKDHSLHLSKAKISKTAPDWEGTAVINGEFKELKL      120
Danio rerio               NGKREQECYNYAGGHVYPGEAFRVPVSDHSLHLSKAKISKPAPHWEGTAVINGEFKELKL      92
Homo sapiens              PRTREEECHFYAGGQVYPGEASRVSVADHSLHLSKAKISKPAPYWEGTAVIDGEFKELKL      103
Xenopus laevis            RTAPDGECHFYAGGQVYPGEASRVPVSDHSLHLSKAKISKPAPYWEGTAVINGEFKELKL      99
Anas platyrhynchos        RQRGDEQCHYYAGGQVYPGEAARLPVTDHSLHLSQAKISKPAPYWEGTAVINGEFKELKL      106
                                       .             .* :: :.* *** ** *:***:::*::: :

Ciona robusta             GDYKGKYLIFFFYPLDFTFVCPTEIIAFSDRVAEFKKINAEVVAASVDSHFTHLAWLNTH      125
Botrylloides leachii      DDYLGKYLIFFFYPLDFTFVCPTEIIAFSDRAEEFRKIGAEVVACSVDSHFTHLAWTNTE      127
Scylla paramamosain       TDYRGKYLVFFFYPLDFTFVCPTEILAFNDRVEEFRALNAEVVACSIDSHFTHLAWTSTP      136
Sepiella maindroni        RSYKGKYLVFFFYPLDFTFVCPTEIIAFSDRIEEFKDLNTKVVACSVDSQFTHLAWLNTP      136
Mus musculus              TDYRGKYLVFFFYPLDFTFVCPTEIIAFGDRIEEFKSINTEVVACSVDSQFTHLAWINTP      166
Python bivittatus         TDYEGKYVVFFFYPLDFTFVCPTEIIAFSDRIEEFRAIDAEVVACSVDSQFTHLAWINTP      180
Danio rerio               SDYKGKYLVFFFYPLDFTFVCPTEIIAFSDRVHEFQAINAEVVACSVDSQFTHLAWINTP      152
Homo sapiens              TDYRGKYLVFFFYPLDFTFVCPTEIIAFGDRLEEFRSINTEVVACSVDSQFTHLAWINTP      163
Xenopus laevis            TDYKGKYLVFFFYPLDFTFVCPTEIIAFGDRIEEFRSINTEVVACSVDSQFTHLAWINTP      159
Anas platyrhynchos        TDYEGKYLVFFFYPLDFTFVCPTEIIAFSDRIEEFRAINTEVVACSVDSKFTHLAWINTP      166
                           .* ***::****************:**.**  **: :.::***.*:**:****** .*

Ciona robusta             RSEGGLGKLNIPLLSDLTHKISRDYGVLLEDLGHTLRGLFIIDPKGILRQITMNDLPVGR      185
Botrylloides leachii      RKEGGLGKINIPLLSDLTHKISRDYGVLLEDLGHTLRGLFIIDPKGILRQITMNDLPVGR      187
Scylla paramamosain       RKDGGLGKLKIPLLSDITHKISKDYGVYLEDQGIALRGLFIIDNKGVLRQITMNDLPVGR      196
Sepiella maindroni        RNKGGLGHLKIPLLSDITHEISKAYGVYLQNLGHSLRGLFIIDTKGILRQITMNDLPVGR      196
Mus musculus              RRQGGLGPIRIPLLSDLNHQISKDYGVYLEDSGHTLRGLFIIDDKGVLRQITLNDLPVGR      226
Python bivittatus         RKQGGLGPVKIPLLSDLTHQISKDYGVYLEDQGHALRGLFIIDDKKILRQITMNDLPVGR      240
Danio rerio               RKQGGLGPMKIPLLSDLTHQISKDYGVFLEDQGHTLRGLFIIDGKGVLRQITMNDLPVGR      212
Homo sapiens              RRQGGLGPIRIPLLSDLTHQISKDYGVYLEDSGHTLRGLFIIDDKGILRQITLNDLPVGR      223
Xenopus laevis            RKQGGLGPMKIPLLSDLTHQISKDYGVYLEDQGHTLRGLFIIDDKGVLRQITMNDLPVGR      219
Anas platyrhynchos        RKQGGLGPMKIPLLSDLTHQISKDYGVYLEDQGHTLRGLFIIDDKRILRQITMNDLPVGR      226
                          * .**** :.******:.*:**: *** *:: * :******** * :*****:*******

Ciona robusta             SVDETLRLVQAFQHTDEHGEVCPA-GWEPGKDTIIPDPKDKLKYFHKTSKAKGEL      239
Botrylloides leachii      SVDETLRLVQAFQHTDQHGEVCPA-GWQPGSDTIIPDPEGKMKYFQKMREL----      237
Scylla paramamosain       SVDETLRLVQAFQFTDEHGEVCPA-GWKPGDDTIIPNPQEKLKYFKKANQ-----      245
Sepiella maindroni        SVDETLRLVQAFQYTDSHGEVCPA-GWKPGADTIIPDPKDKLKYFEKANS-----      245
Mus musculus              SVDETLRLVQAFQYTDKHGEDNPRSSWKTEVFRQAKLKSTSVMMFGPSQ------      275
Python bivittatus         SVDETLRLVQAFQYTDKHGEVCPA-GWKPGSETIIPDPAGKLKYFGKVN------      288
Danio rerio               SVDETLRLVQAFQYTDKHGEVCPA-GWKPGSDTIIPDPAGKLKYFDKLN------      260
Homo sapiens              SVDETLRLVQAFQYTDKHGEVCPA-GWKPGSETIIPDPAGKLKYFDKLN------      271
Xenopus laevis            SVDETLRLVQAFQYTDKHGEVCPA-GWKPGSETIIPDPAGKLKYFDKQH------      267
Anas platyrhynchos        SVDETLRLVQAFQYTDKHGEVCPA-GWKPGSETIIPDPAGKLKYFDKLN------      274
                          *************.**.***  *  .*:            .:  *
```

Figure S6. Schematic Prdx4 domain organization. The AhpC-TSA domain is highlighted in light grey, the 1-cysPrx_C domain is highlighted in dark grey. For *C. robusta*, alpha helixes and beta sheets are boxed with dotted and continues lines respectively; amino acids of the catalytic center are indicated in bold and underlined. *= identical amino acids (completely preserved), := very similar amino acids (semi-conservative substitution), .= similar amino acids (conservative substitution); numerals refer to character counts.

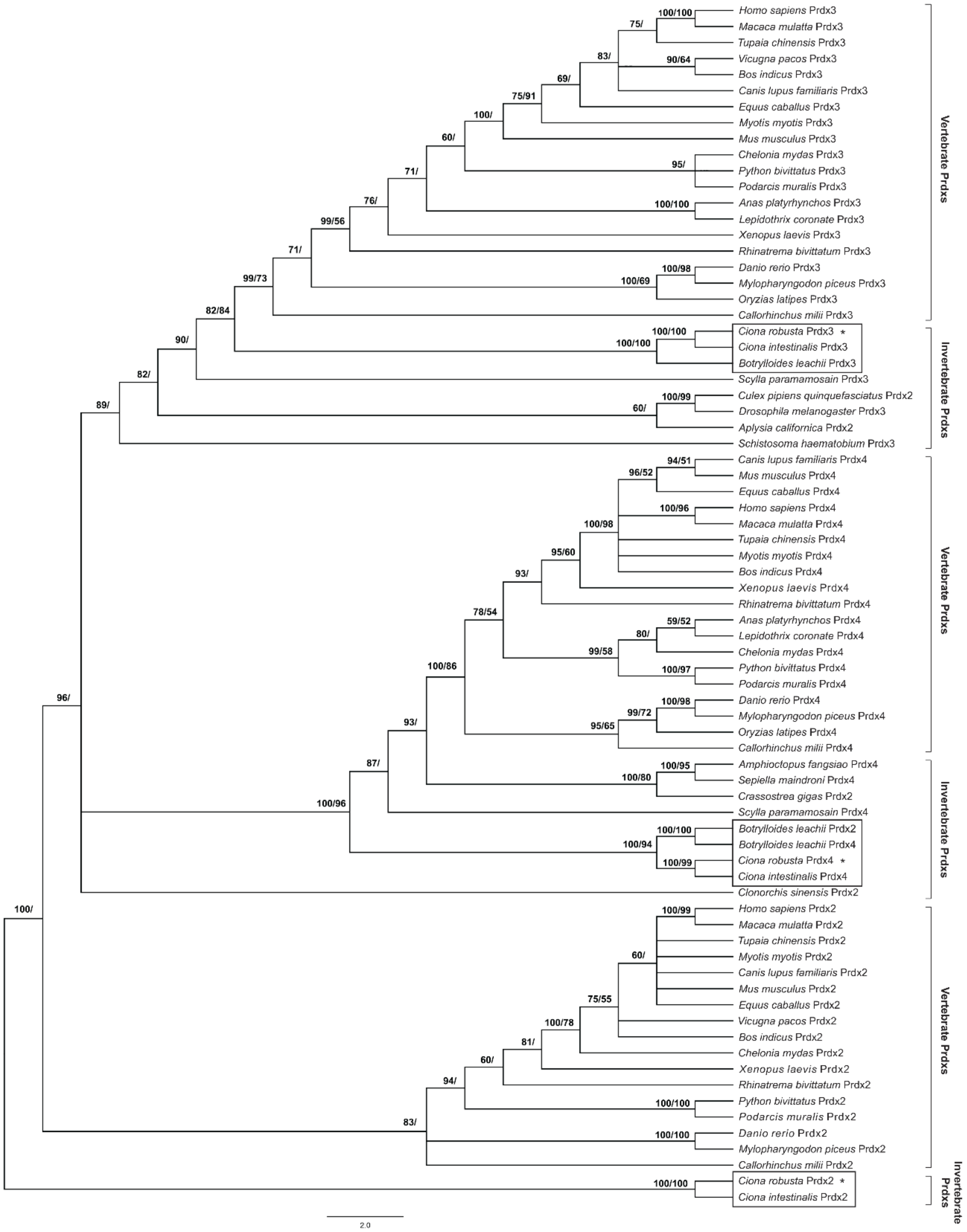

Figure S7. Phylogenetic relationships among Prdx isoforms of various organisms reconstructed on the amino acid sequences and using both Bayesian interference (BI) and Maximum likelihood (ML) methods. Bayesian posterior probability (first number) and bootstrap values higher (and equal to) than 50% are indicated on each node, respectively. The scale for branch length (2.0 substitution/site) is shown below the tree.
